# Recycling-Oriented Development and Microstructure–Property Evaluation of High-Recycled 6xxx Aluminum Alloys and CRM-Lean 6111 Alloy for Automotive Applications

**DOI:** 10.3390/ma19020377

**Published:** 2026-01-17

**Authors:** Zeynep Tutku Ozen, Necip Unlu, Irem Yaren Siyah, Sonia Boczkal, Gorkem Ozcelik, Salim Aslanlar

**Affiliations:** 1 Chemistry and Metallurgy Faculty, Metallurgical and Materials Engineering Department, Istanbul Technical University, 34467 Istanbul, Türkiye; unlu@itu.edu.tr; 2 ASAS Aluminyum A.S., 34810 Istanbul, Türkiye; yaren.siyah@asastr.com; 3 Lucasiewicz Research Network—Institute of Non-Ferrous Metals, 44-100 Gliwice, Poland; sonia.boczkal@imn.lukasiewicz.gov.pl; 4 Manufacturing Engineering Department, Sakarya Applied Sciences University, 54050 Serdivan, Türkiye; gorkem.ozcelik@asastr.com (G.O.); aslanlar@subu.edu.tr (S.A.)

**Keywords:** 6xxx alloys, recycling, post-consumer scrap, extrusion, homogenization, casting, SEM, TEM, EBSD, mechanical properties

## Abstract

**Highlights:**

**What are the main findings?**

- MULTI-PICK–sorted aluminum scrap enables high melt cleanliness suitable for extrusion.
- Detailed OM, SEM/EDS, TEM, and EBSD analyses reveal that 6063 and 6082 alloys with high recycled content develop stable and uniform microstructures.
- The high-recycled 6063 and 6082 alloys, as well as the CRM-lean 6111 alloy, meet the mechanical requirements for automotive applications.

**What is the implication of the main finding?**

- High post-consumer scrap content can be safely integrated into 6xxx aluminum alloys without compromising performance.
- Advanced sorting and controlled processing enable reliable industrial use of recycled aluminum for structural automotive components.
- The results support scalable, circular-economy aluminum production aligned with sustainability targets.

**Abstract:**

Recycling of 6xxx aluminum alloys, which are used extensively in the automotive industry, is important for ensuring a carbon-neutral future and the efficient use of resources on Earth. The sustainability of recycling in aluminum alloys is directly proportional to the correct classification of the scrap to be used. In this study, scrap stream from a novel scrap-sorting technology called MULTI-PICK has been used to validate. The 6063 and 6082 alloys produced with scrap stream, which are commonly used for structural parts in the automotive sector, are analyzed with hydrogen analysis and PREFIL. Cast billets are evaluated considering extrusion. After extrusion, microstructures of the profiles are investigated with scanning electron microscopy (SE), transmission electron microscopy (TE) and electron backscatter diffraction (EBSD). Their mechanical properties and anisotropic behaviors are investigated with tensile testing in different orientations. Additionally, an alternative alloy called 6111 has been studied to replace the target alloys with low critical raw material (CRM) content. According to the findings, highly recycled 6xxx alloys can be used in the automotive industry without losing their existing properties. Furthermore, using alternative feedstock and retrofitted systems can decrease carbon footprint below 4 kgCeq/kgAl.

## 1. Introduction

Environmental, economic and energy-related reasons make aluminum recycling an important research area. The first reason is the conservation of natural resources. The primary raw material of metallic aluminum is bauxite ore, which is finite. The extraction and processing requirements are reduced because of the increased recycling applications of aluminum. In addition, the processing of bauxite ore through the Bayer process and molten salt electrolysis requires a significant energy input. It is reported that recycling aluminum saves up to 95% of the energy required for primary production. Moreover, primary aluminum production is one of the main sources of the greenhouse gas emissions causing climate change. Recycling aluminum helps to significantly reduce these emissions. Lastly, aluminum is a highly durable and versatile material, which can be used repeatedly without compromising its quality. This extends the life cycle of products and reduces the need for frequent manufacturing [1,2].

Bauxite ore, Mg and Si are listed among the critical raw materials (CRMs) defined by the European Commission (EC), due to their economic importance and supply risk. These CRMs are required to produce 6xxx aluminum alloys, which are commonly used in the automotive sector. The EC promotes alternative solutions such as increasing recycling rates and reducing dependency on CRMs by substituting them with more abundant elements. This study supports the European Green Deal goals and circular economy principles by proposing CRM-lean aluminum alloys that are suitable for electric vehicle (EV) applications [3,4,5,6].

Moreover, the influence of impurities—especially Fe and other residual elements—on the microstructural integrity and mechanical behavior of scrap-based alloys remains a critical concern. Recent work on the recycled 6111 alloy revealed that elevated Fe content leads to an increased density of Fe-rich intermetallic compounds (IMCs) and significantly altered damage and fracture behavior under tensile loading [7].

Given these challenges, there is a clear need for systematic studies that integrate the entire recycling-to-product chain: from advanced scrap sorting, melt quality control, casting and billet quality inspection, homogenization and microstructural homogenization, to final extrusion, microstructure characterization (SEM, TEM, EBSD) and mechanical testing. In doing so, one can rigorously assess whether high-recycled-content 6xxx alloys can truly meet industrial requirements without performance loss and under determined processing conditions.

While previous studies have demonstrated the potential to incorporate scrap into 6xxx-series alloys with acceptable performance, many have focused only on limited aspects—for example, melt quality or mechanical properties—without comprehensive tracking of the full process route from scrap sorting to microstructure and final mechanical performance. For instance, experimental work on recycled 6000-series sheet production showed that even with a high scrap content, the microstructure and tensile properties remained similar to the primary material [8]. Another study has demonstrated the feasibility of producing high-performance recycled alloys for aerospace applications [9]. These studies largely focus on wrought-sheet processing and single-alloy systems. In contrast, this work expands the recycling framework to multiple 6xxx alloys, integrates industrial-scale extrusion validation and evaluates full process-chain behavior from scrap sorting to mechanical anisotropy.

In this context, the present study aims to demonstrate a carefully controlled process route. This study involves advanced scrap sorting (MULTI-PICK), melt quality assessment (hydrogen measurement and inclusion analysis via PREFIL), ultrasonic billet control after casting, microstructural inspection before and after homogenization via optical microscopy and comprehensive post-extrusion microstructure (SEM, TEM, EBSD) and mechanical testing of high-recycling-rate 6063 and 6082 alloys and CRM-lean 6111 alloy that meet the mechanical and structural demands of automotive applications. By doing so, this work not only contributes to the sustainable alloy design and circular economy but also bridges the gap between laboratory-scale recycling studies and industrially relevant validation.

## 2. Materials and Methods

**Figure 1:**
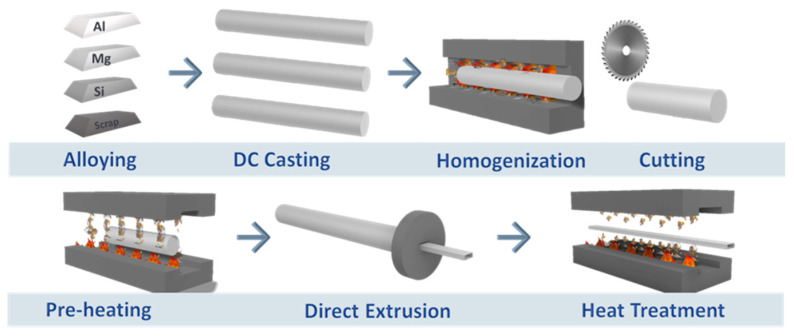
Production route.

There are two different strategies adapted in this study to limit critical raw material (CRM) usage. The first one is decreasing the Mg and Si ratios in the 6xxx alloy without compromising the desired properties. To achieve that, a new alloy, 6111, has been developed. The second strategy is supplying CRMs, Mg and Si, from post-consumer scraps in 6xxx alloys. The 6063 and 6082 alloys have been selected for this approach, since they are some of the most common alloys used in the automotive industry. In this study, two complementary low-CRM strategies were investigated within the 6xxx alloy family. The first approach focused on reducing critical raw material usage through alloy design, represented by the CRM-lean 6111 alloy produced from primary feedstock with minimized Mg and Si additions. The second approach relied on substituting critical raw materials via high post-consumer scrap utilization, which was implemented for the 6063 and 6082 alloys. This dual strategy enables a systematic comparison between alloy design-based and recycling-based pathways toward low-CRM aluminum production. The production route for the alloys has been demonstrated in Figure 1. The 6063 and 6082 alloys’ production route begins with an advanced sorting technology of the post-consumer aluminum scraps called MULTI-PICK, developed by University of Liege MAT-VISION team. This sorting technology combines several sensor technologies (spectral, 3D, LIBS, etc.) to discriminate between the different alloys and separate them using ejectors or gripper robots (MAT-VISION, Liege, Belgium) [10]. Scraps sorted by MULTI-PICK integrated with the COMET facilities in Belgium have been transferred to IMN facilities in Poland for lab-scale validations and to ASAS Aluminum in Turkiye for industrial validations. In the trials, sorted post-consumer scraps are used both for lab-scale and industrial trials, starting from melting and then alloying.

The molten aluminum alloys were produced by DC casting, using Wagstaff AirSlip technology (Wagstaff Inc., Washington, DC, USA). Table 1 summarizes the feedstock composition of the investigated alloys. The 6082 alloy was produced using 100% post-consumer scrap, while the 6063 alloy was produced using a mixture of 70% post-consumer scrap and 30% pre-consumer scrap. The 6111 alloy was produced entirely from primary feedstock; however, its chemical design was optimized to limit the usage of critical alloying elements such as magnesium and silicon.

Table 2 demonstrates the chemical compositions of the alloys. The chemical compositions of the alloys were determined by using optical emission spectroscopy (OES). Measurements were carried out on solid samples taken from liquid metal flow during casting, using a calibrated OES spectrometer Thermo Fisher Scientific brand ARL 3460 model (Thermo Fisher Scientific, Waltham, MA, USA). Multiple measurements were performed for each alloy to ensure compositional homogeneity and accuracy. Special attention was given to the determination of the Fe and Si contents, due to their critical role in recycled aluminum alloys and their strong influence on microstructural evolution and mechanical properties. The 6063 and 6082 alloys are controlled with respect to the related alloy standard, EN-573-3 [11]. It is seen that both compositions of these alloys are in the standard range.

**Figure 2:**
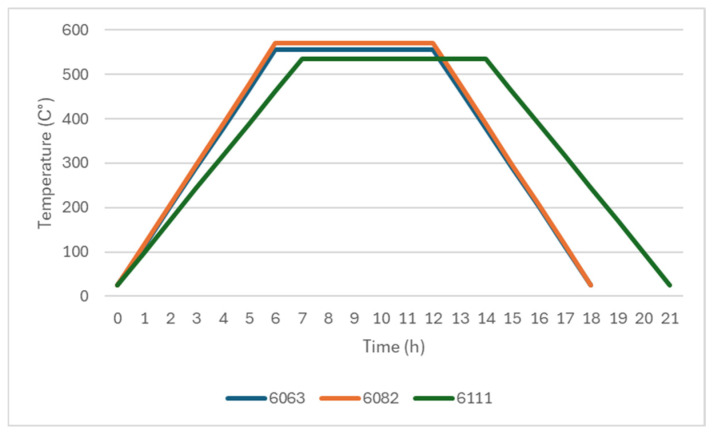
Homogenization practice of the alloys.

Cast billets underwent a homogenization procedure to be ready for the extrusion step in a batch-type homogenization furnace. Figure 2 shows the homogenization graph for the alloys. Graph shows that the 6063 billets were heated up to 550–570 °C from room temperature in 6 h. They were kept at 550–570 °C for 6 h and cooled down to room temperature in 6 h. The 6082 billets were heated up to 560–580 °C from room temperature in 6 h. They were kept at that temperature for 6 h and cooled down to room temperature in 6 h. Finally, the 6111 billets were heated up to 530–540 °C from room temperature in 7 h. They were kept at 530–540 °C for 7 h and cooled down to room temperature in 7 h.

After homogenization, the aluminum billets were ready for extrusion. Billets were cut to an 800–1100 mm length and pre-heated to 480–500 °C for the extrusion process. Two types of geometries were selected to validate both the structural and battery box parts. A 62 MN capacity extrusion press was used to extrude the selected profiles. The extrusion speed was selected between 4.5 and 7.5 mm/s. All the profiles were quenched with 100% water spray. Heat treatment was applied to profiles after extruding and quenching.

Along with production, material characterization methodologies were applied to sustain deeper investigation into recycling metallurgy. PREFIL (ABB Measurement & Analytics, Zürich, Switzerland) and hydrogen analysis were applied to molten aluminum to control the metal quality. Molten aluminum of the selected alloys was analyzed with an AlScan hydrogen content analyzer (ABB Measurement & Analytics, Zürich, Switzerland), according to EN 583 and EN 12680-1 standards [12,13]. Ultrasonic inspection was applied to cast billets to detect internal defects such as cracks and voids prior to extrusion. The testing was carried out using a pulse-echo ultrasonic testing method, in accordance with ASTM B594 [14]. The inspection ensured billet integrity and extrusion feasibility. Both the homogenized and non-homogenized billets were controlled by optical microscopy. After extrusion and heat treatment, tensile strength samples were prepared from the profiles. They were controlled by SEM and TEM. Metallographic samples were sectioned from billets and extruded profiles, mounted, ground and polished, using standard metallographic procedures. The final polishing was performed using colloidal silica suspension to obtain a deformation-free surface that was suitable for microstructural analysis. Optical microscopy was conducted to evaluate the grain structure and intermetallic phase distribution, applying etching with 600 mL HNO_3_ (ACS reagent grade, Sigma-Aldrich, St. Louis, MO, USA), 200 mL HCl ((37%, Merck KGaA, Darmstadt, Germany)), 10 mL HF (48%, Merck KGaA, Darmstadt, Germany) and 350 mL pure water solution. Scanning electron microscopy (SEM) was used for detailed microstructural examination, including the morphology and distribution of secondary phases. An SEM device Zeiss EVO MA15(ZEISS, Oberkochen, Germany) was used with 20 kV energy back scattered electrons, a 60 mm working distance and 3000× magnification. EDS analysis has been carried out for specified points and areas from SEM images, using the ZEISS SmartEDX(ZEISS, Oberkochen, Germany) attachment on the SEM device. TEM analysis has been carried out to extrusion profiles. All the alloy variants were polished electrochemically. A FEI TecnaiG2 20XT (Thermo Fisher Scientific, Hillsboro, OR, USA) (200 kV) high-resolution transmission electron microscope equipped with STEM and EDX was used to take bright-field images. EBSD analysis of the samples was carried out after polishing them using conventional metallographic methods; they were exposed to ion etching. Utilizing a high-resolution INSPECT F50 (Thermo Fisher Scientific, Hillsboro, OR, USA) scanning electron microscope with the camera Velocity plus for EBSD, the crystallographic orientation was performed. EBSD data were acquired by using an SEM equipped with an EBSD detector, operated at an accelerating voltage of 20 kV and a step size of 1 µm. Orientation maps were analyzed to evaluate grain size, crystallographic texture and local misorientation. Tensile tests were conducted on specimens machined from extruded profiles, according to the relevant standards. Uniaxial tensile tests were performed by using a ZwickRoell Z050 (Zwick Roell GmbH & Co. KG, Ulm, Germany) tensile testing machine with a load capacity of 100 kN, in accordance with EN ISO 6892-1:2020 [15]. The specimen geometry and test parameters were defined based on the same standard, and a constant crosshead speed of 10 mm/min was applied. The tensile strength, yield strength and elongation were determined, and anisotropy was evaluated by testing samples extracted at different orientations, relative to the extrusion direction.

## 3. Results

### 3.1. Molten Metal Analysis Results

#### 3.1.1. Hydrogen Content Analysis Results

**Figure 3:**
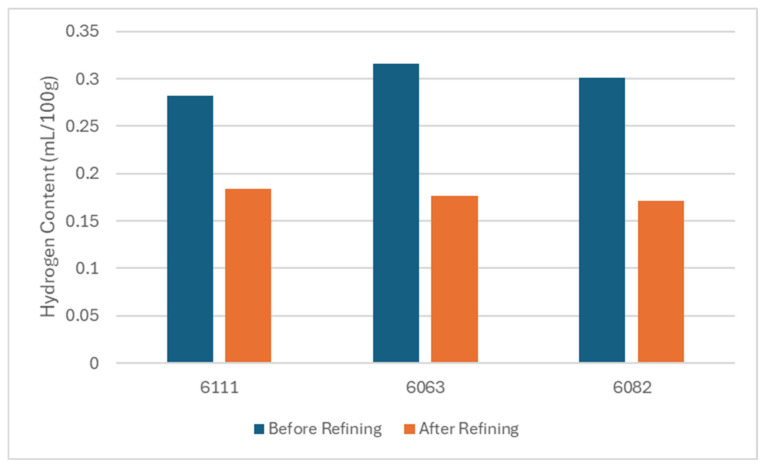
Hydrogen content analysis during casting.

Figure 3 demonstrates the hydrogen content analysis results for the 6111, 6063 and 6082 alloys during casting and before and after the refining process. The hydrogen content of the 6111 alloys has decreased from 0.28 mL/100 g to 0.18 mL/100 g after rafination. The hydrogen content of the 6063 alloys has decreased from 0.32 mL/100 g to 0.17 mL/100 g after rafination. The hydrogen content of the 6082 alloys has decreased from 0.3 mL/100 g to 0.17 mL/100 g after rafination. According to a study of Samuel et al., a hydrogen content over 0.25 mL/100 g is considered to be a high hydrogen content for aluminum alloys. Casting of the molten aluminum with a high hydrogen content may yield gas porosity formation and therefore create discontinuity and tearing potential during the extrusion process [16].

#### 3.1.2. Inclusion Analysis via PREFIL Results

**Figure 4:**
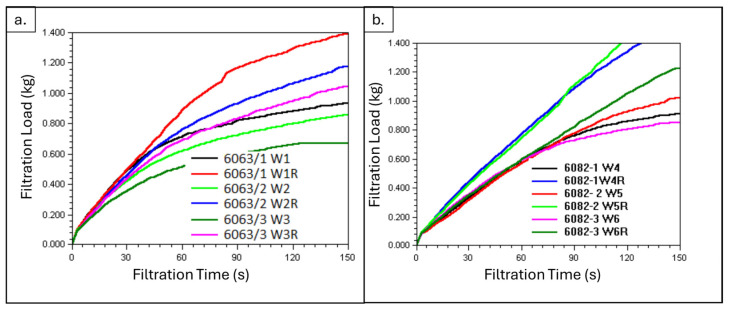
Filtration load–time curves obtained from PREFIL^®^ inclusion analysis of molten aluminum alloys. (**a**) 6063 alloy and (**b**) 6082 alloy.

Inclusions of the molten metal were analyzed using a PREFIL^®^ instrument before and after refining. Inclusion analyses were only applied to the 6063 and 6082 alloys, since the 6111 alloy is not produced by using scrap or secondary ingot. Figure 4 demonstrates the curves of weight–time for three samples taken before (W1, W2, W3, W4, W5 and W6) and after (W1R, W2R, W3R, W4R, W5R and W6R) refining these two alloys with the PREFIL^®^ software. (Prefil^®^ software and Prefil^®^ Database Software) A low inclusion content yielded a fast increase in terms of weight in the PREFIL^®^ analysis. It can be seen that refined samples had a lower inclusion content compared to their before-refining samples.

The residues of the molten metal taken from the filter of this analysis were prepared for metallographic analysis. The content of the inclusions concentrated on the surface of the test filter is then quantified, using image analysis software (ZEN Blue imaging software (Carl Zeiss Microscopy GmbH, Jena, Germany). LM (Ziess Axio Observer) images with a total area of 0.6 mm^2^ for each sample underwent quantitative analysis. This was then normalized by both the nominal chord length and the mass of filtered metal to give the familiar units of mm^2^/kg. Inclusions were arbitrarily classified by class and content (in mm^2^/kg). According to a study carried out by Stanica et al., inclusion concentrations are specified as very light if the value is between 0 and 0.05 mm^2^/kg, light if the value is between 0.05 and 0.1 mm^2^/kg, moderate if the value is between 0.1 and 0.4 mm^2^/kg, high if the value is between 0.4 and 1.2 mm^2^/kg and very high if the value is over 1.2 mm^2^/kg [17].

**Figure 5:**
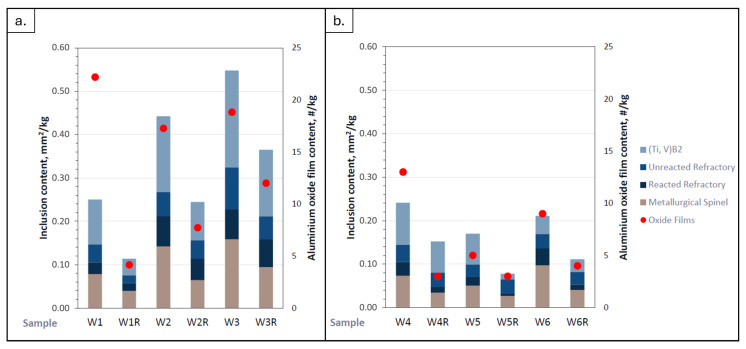
Inclusion content and aluminum oxide index obtained from PREFIL^®^ analysis for different sample designations. (**a**) 6063 alloy and (**b**) 6082 alloy.

Figure 5 presents the results of the inclusion analysis. The main types of inclusions identified were metallurgical spinels, refractory particles (both reacted and unreacted), boron treatment residues such as (Ti,V)B_2_ and aluminum oxide films. After the refining process, the total inclusion content (TIC) showed a noticeable reduction. The refined 6063 alloy sample (first sample) exhibited the lowest TIC value of 0.113 mm^2^/kg, placing it in the moderate inclusion class. Metallographic examination of the inclusions located above the filter in the 6063 alloy samples analyzed with the PREFIL device revealed that the predominant fraction consisted of (Ti,V)B_2_ particles. For the 6082 alloy, the fraction of metallurgical spinels decreased by approximately half, and the reacted refractory particles were reduced by nearly threefold, while unreacted refractory inclusions remained constant at about 0.03–0.04 mm^2^/kg. The lowest TIC value, 0.078 mm^2^/kg, was observed in the W5R sample, corresponding to the light inclusion class. The other samples were categorized within the moderate inclusion class.

### 3.2. Extrusion Feasibility Analysis Results

#### 3.2.1. Ultrasonic Testing Results

After casting, the billets are subjected to ultrasonic examination to assess internal defects such as cracks or inclusions that could negatively affect the extrusion performance and final product quality. Ultrasonic inspection is a widely used non-destructive testing method for detecting internal discontinuities in aluminum billets. The results of the ultrasonic analysis carried out with respect to the ASTM B 594 standards met the acceptance criteria and were deemed suitable for subsequent processing.

#### 3.2.2. Microstructure Investigation via Optical Microscopy Results

**Figure 6:**
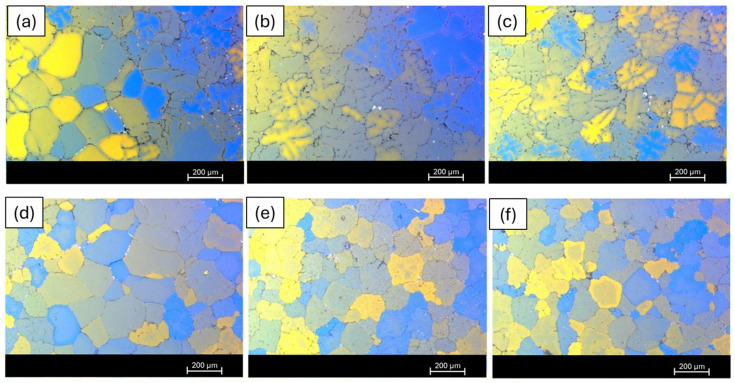
Optical image of 6063 billets before homogenization—(**a**) central zone, (**b**) quarter zone (middle) and (**c**) shell zone—and after homogenization: (**d**) central zone, (**e**) quarter zone (middle) and (**f**) shell zone.

A metallographic analysis was conducted in the billets in order to evaluate the differences between the inner and the outer regions of the billet. Figure 6a–c illustrate the optical micrographs captured from the central, quarter and shell regions of the 6063 billets prior to the homogenization procedure. As anticipated, a gradual coarsening of the aluminum matrix grains is observed from the outer shell towards the central region, which is consistent with the local solidification condition.

In Figure 6d–f, optical images of the central zone, quarter zone and shell zone of the 6063 billet after homogenization are shown. The microstructure reveals a similar tendency as that observed before the homogenization treatment (Figure 6a–c), consistent with the previous observation. The optical micrographs indicate a more uniform and homogenized microstructural appearance after homogenization treatment. The aluminum matrix exhibits improved continuity, and no pronounced microstructural heterogeneity is observed at the presented magnification level. The observed changes are therefore discussed in terms of overall microstructural uniformity, rather than specific phase-related features. These observations strongly suggest that the homogenization process was applied correctly and effectively.

**Figure 7:**
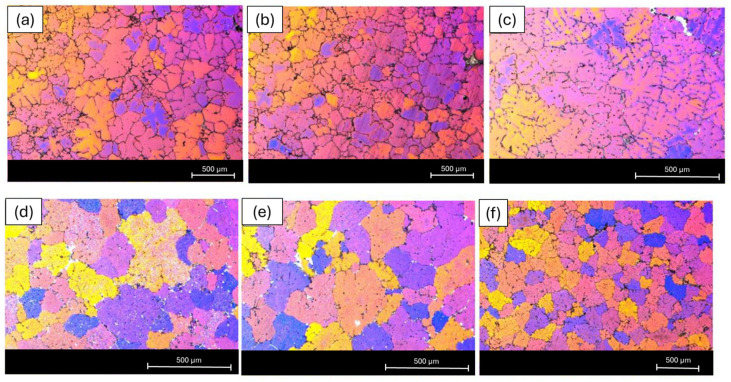
Optical image of 6082 billets, before homogenization—(**a**) central zone, (**b**) quarter zone and (**c**) shell zone—and after homogenization: (**d**) central zone, (**e**) quarter zone and (**f**) shell zone.

Similar investigations were conducted on the 6082 alloy. Figure 7a–c illustrate the central, quarter and shell zones of the 6082 alloy prior to homogenization. Upon comparison with the 6063 alloy, it is observed that the average grain size in the 6082 alloy is comparatively smaller. Additionally, due to the higher presence of alloying elements in the 6082 alloy, the density of intermetallic (secondary phases) is also higher. Post-homogenization images are provided in Figure 7d–f. Here, compared to the pre-homogenization images, secondary phases are more uniformly distributed, and the grains exhibit a more regular pattern. A reduction in the segregation amount is also observed. The outcomes obtained from these mechanisms have a strong resemblance to those observed in the 6063 alloy.

**Figure 8:**
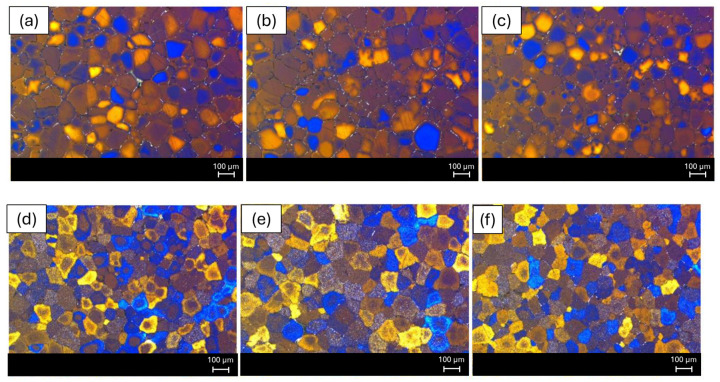
Optical image of 6111 billets before homogenization—(**a**) central zone, (**b**) quarter zone and (**c**) shell zone—and after homogenization: (**d**) central zone, (**e**) quarter zone and (**f**) shell zone.

Figure 8a–c illustrate the microstructure of the 6111 billet before homogenization. Compared to the 6063 and 6082 alloys, the 6111 alloy contains a significantly higher proportion of alloying elements, particularly copper. Figure 8a–c illustrate the typical as-cast microstructural features of the 6111 billet, which are characterized by equiaxed aluminum grains across the central, quarter and shell regions. Figure 8d–f present the post-homogenization image of the 6111 billet. It is clearly observed that the grains, intermetallics and precipitates are distributed homogeneously compared to the before homogenization status of the exact same billet.

Through the homogenization process, the ß-AlFeSi intermetallic phases underwent a transformation into α-AlFeSi, thereby mitigating the potential risk of localized melting during extrusion. Moreover, within the 6xxx series alloys, the presence of Mn, Cr and Zr particles required homogenization, due to their propensity for precipitation at elevated temperatures [18]. Comparative analysis of pre- and post-homogenization microstructures reveals the attainment of adequate homogenization: a crucial observation aligning with subsequent extrusion outcomes.

Furthermore, upon examination of the shell, quarter and central zones of the cast billets, it was noted that the intermetallic phases and segregated regions were predominantly situated nearer to the shell zone, as expected. This observation persists even after the homogenization process, which facilitates micro-segregation-level uniformization. However, it is noteworthy that these regions exhibit acceptable characteristics. Notably, during the extrusion process, most of the shell area is typically discarded.

### 3.3. Microstructure and Performance Analysis Results of the Aluminum Profiles

#### 3.3.1. Scanning Electron Microscopy (SEM) and Energy Dispersive Spectrometer (EDS) Results

**Figure 9:**
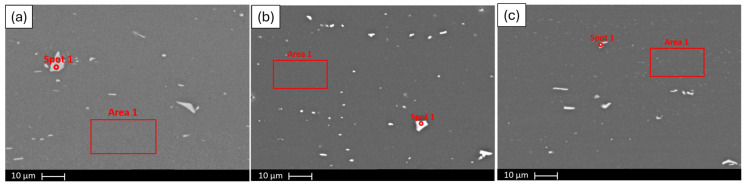
SEM images under 3000× magnification of (**a**) 6063 alloy profile, (**b**) 6111 alloy profile and (**c**) 6082 alloy profile.

Figure 9a–c demonstrate the 6063, 6111 and 6082 alloy profile SEM images under 3000× magnification. Secondary phases are observed as white marks in these images. The SEM images suggest that secondary-phase particles in the 6063 and 6082 alloys appear coarser and more heterogeneously distributed, whereas the 6111 alloy exhibits finer and more uniformly distributed particles.

Energy-dispersive X-ray spectroscopy (EDS) analyses were performed on the bright contrast regions observed in the SEM images to investigate their chemical composition. The corresponding EDS results are summarized in Table 3. For the 6063 alloy profile, the white contrast regions were found to be enriched in Al, Fe and Si, indicating the presence of Fe–Si-rich secondary phases within the aluminum matrix. In the case of the 6111 alloy, EDS analyses revealed that the bright regions contained Al, Fe, Mn and Si, with selected spot analyses also showing a noticeable presence of Cu. This is consistent with the higher Cu content of the 6111 alloy. Similarly, the EDS results obtained from the 6082 alloy profile indicate that the white contrast regions are enriched in Al, Fe, Mn and Si.

Table 4 shows the EDS analysis performed to the marked area and spot of Figure 9b.

#### 3.3.2. Transmission Electron Microscopy (TEM) and Electron Dispersive Spectrometer (EDS) Results

**Figure 10:**
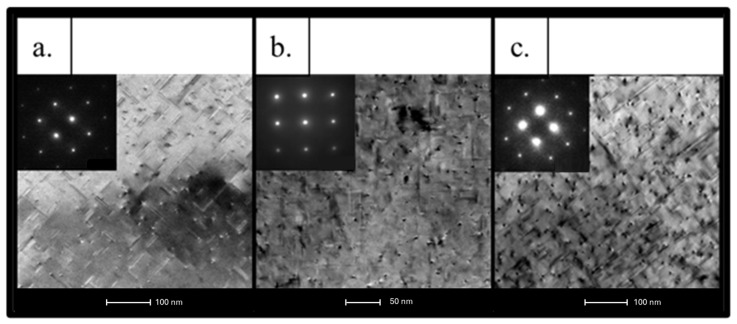
TEM bright-field images: (**a**) 6063 profile; (**b**) 6111 profile; and (**c**) 6082 profile.

The bright-field TEM images shown in Figure 10a–c reveal a well-defined aluminum matrix with pronounced dislocation structures in all investigated alloys, while the corresponding SAED patterns indicate a crystalline matrix without evidence of amorphization. The images reveal the presence of fine nanoscale contrast features within the aluminum matrix, which are indicative of the microstructural heterogeneities formed during processing. A comparison among the alloys shows that the 6063 and 6082 profiles exhibit relatively coarser and more heterogeneous contrast features, whereas the 6111 alloy displays finer and more uniformly distributed nanoscale features. Although the exact nature and chemistry of these features cannot be conclusively identified based solely on bright-field TEM observations, the observed differences in size and distribution suggest a more homogeneous microstructural state in the 6111 alloy. Such a refined and uniform nanoscale distribution has been reported in the literature to be favorable for mechanical performance, which may contribute to the higher strength levels observed in the 6111 alloy [19]. Considering the lower Fe content of the 6111 alloy, as indicated by its chemical composition, the absence of coarse nanoscale features may be associated with a reduced tendency for the formation of Fe-rich microstructural heterogeneities [20,21].

**Figure 11:**
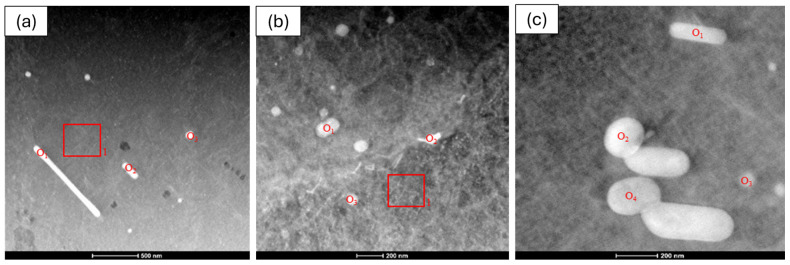
TEM images of (**a**) 6063 alloy profile, (**b**) 6111 alloy profile and (**c**) 6082 alloy profile. Where O’s stands for points and rectangles stands for the area that the measurement taken.

The bright-field TEM images shown at Figure 11a–c indicate the presence of nanoscale contrast features with varying morphologies across the investigated alloys. The 6063 and 6082 profiles exhibit relatively coarser (100 nm–1 µm for 6063 and 150–400 nm for 6082) and more heterogeneous features, including elongated and irregularly shaped contrasts, whereas the 6111 alloy displays finer (100–200 nm) and more uniformly distributed nanoscale features. Although the exact nature of these features cannot be conclusively identified based solely on bright-field TEM observations, the observed differences suggest a more homogeneous microstructural state in the 6111 alloy. Such a refined nanoscale distribution has been reported to be favorable for mechanical performance in Al–Mg–Si alloys [22].

Three spot analyses taken from the bright contrast regions and one area analysis from the matrix were chemically examined by EDS. The results are summarized in Table 4. The spot analyses (O_1_–O_3_) reveal that the bright regions are enriched in alloying elements such as Fe, Mn and Si, with a minor presence of Cu, indicating the formation of Fe-containing secondary phases within the aluminum matrix. In contrast, the area analysis (Area 1) is dominated by aluminum, confirming that it represents the matrix region.

EDS analyses were performed on two representative bright contrast regions and one matrix area in the 6111 alloy profile, and the results are summarized in Table 5. The spot analyses reveal that the bright regions are enriched in alloying elements such as Mn and Si, with a minor presence of Cu, while the Fe content remains relatively low. In contrast, the area analysis is dominated by aluminum, confirming that it corresponds to the matrix region. These results indicate that the secondary phases observed in the 6111 alloy are primarily Mn- and Si-rich, with limited Fe contribution, which is consistent with the lower Fe content of this alloy.

EDS analyses were conducted on four representative bright contrast regions in the 6082 alloy profile, and the results are summarized in Table 6. The spot analyses indicate that the bright spots are enriched primarily in Mn and Si, with minor contributions from Cr, while the Fe content remains relatively low. One of the analyzed points exhibits a composition closer to that of the aluminum matrix, suggesting local compositional variations within the microstructure. Overall, the EDS results indicate that the secondary phases that are present in the 6082 alloy are predominantly Mn–Si rich with limited Fe contribution.

#### 3.3.3. Electron Backscatter Diffraction (EBSD) Results

**Figure 12:**
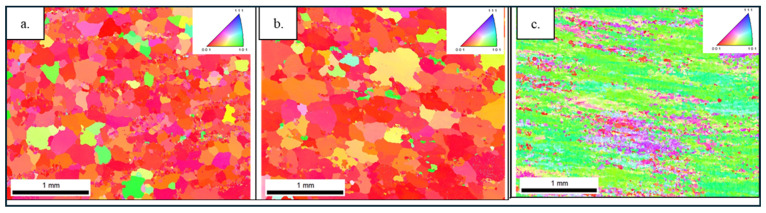
Inverse pole figure (IPF) images from EBSD analysis (**a**) 6063; (**b**) 6111; and (**c**) 6082 profiles.

The EBSD inverse pole figure (IPF) maps shown in Figure 12 indicate that the grains in the 6063 and 6111 alloys exhibit a predominant orientation that is close to the ⟨001⟩ direction, whereas the 6082 alloy shows a different preferred orientation, tending towards the ⟨101⟩ direction. Furthermore, the orientation colors in the IPF maps of the 6063 and 6111 alloys appear to be relatively clustered, suggesting a limited spread in crystallographic orientations. Such a relatively narrow orientation distribution has been reported in the literature to be associated with improved deformation uniformity and favorable extrusion behavior in aluminum alloys. In contrast, the elongated and banded orientation features observed in the 6082 alloy reflect the deformation-induced texture developed during extrusion.

**Figure 13:**
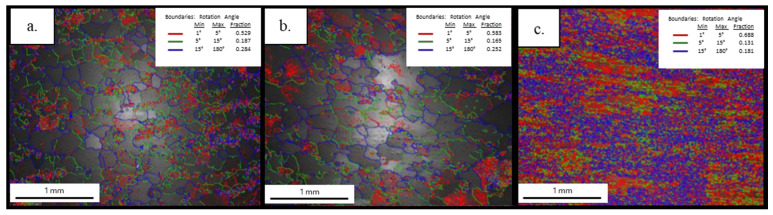
EBSD grain boundary maps showing the distribution of low- and high-angle boundaries in (**a**) 6063, (**b**) 6111, and (**c**) 6082 profiles. Boundaries are classified by misorientation angle as 1°–5° (very low-angle, red), 5°–15° (low-angle, green), and >15° (high-angle, blue). The inset legend reports the minimum (Min) and maximum (Max) misorientation angles defining each class and the corresponding boundary fraction, which represents the relative proportion of total boundary length belonging to that angle range within the scanned area. Scale bar: 1 mm3.3.4. Anisotropic Tensile Strength Analysis Results.

Figure 13 presents EBSD boundary maps distinguishing low-angle grain boundaries (LAGBs, <15°) and high-angle grain boundaries (HAGBs, >15°) for the investigated alloys. Both the 6063 and 6111 alloys exhibit comparable boundary characteristics, with a notable presence of low-angle boundaries, indicating a microstructure dominated by subgrain structures that are typically associated with deformation-induced features. In contrast, the 6082 alloy displays a more heterogeneous boundary distribution with a higher proportion of high-angle boundaries, reflecting a distinctly different grain-boundary character. Such variations in grain-boundary distributions have been reported to influence deformation behavior and texture evolution in aluminum alloys, without implying a fully recrystallized state. Differences in the fraction and distribution of low- and high-angle grain boundaries have been reported to affect strain accommodation and deformation uniformity in aluminum alloys [23,24].

Tensile tests were performed to obtain information about their anisotropic behavior. For each alloy and geometry, 0°, 45° and 90° tensile test samples were prepared according to the extrusion directions and tensile tests were performed. The obtained results are listed in Table 7 for all alloys.

The anisotropic tensile behavior observed in the investigated alloys is primarily governed by the extrusion-induced texture, grain-boundary character distribution and microstructural homogeneity. Previous studies have demonstrated that the strong crystallographic texture developed during extrusion leads to a pronounced directional dependence on yield strength and ductility in Al–Mg–Si alloys [24].

For the 6063 and 6082 alloys, the higher strength values observed in the transverse direction and reduced ductility at 45° indicate a strong texture influence combined with heterogeneous grain boundary characteristics. Such behavior has been reported to be associated with an increased fraction of high-angle grain boundaries and non-uniform strain accommodation during deformation [23].

In contrast, the 6111 alloy exhibits reduced strength anisotropy, which can be attributed to its lower Fe content and more homogeneous microstructure. Recent studies have shown that decreasing the Fe content suppresses the formation of coarse Fe-rich intermetallics, leading to improved deformation uniformity and reduced orientation sensitivity [20].

Furthermore, operando microstructural investigations have highlighted that a refined and uniformly distributed nanoscale microstructure promotes homogeneous plastic deformation and mitigates an anisotropic mechanical response in aluminum alloys. This observation is fully consistent with the present findings for the 6111 alloy, where refined microstructural features contribute to nearly isotropic strength behavior, despite extrusion processing [25].

Overall, the combined effects of texture, grain boundary character distribution and intermetallic morphology govern the anisotropic mechanical response of extruded Al–Mg–Si alloys. Alloys exhibiting refined and homogeneous microstructures demonstrate reduced anisotropy, whereas pronounced texture and heterogeneous boundary distributions enhance orientation-dependent mechanical behavior.

These anisotropic trends are consistent with the EBSD results, which revealed pronounced extrusion-induced texture in the 6063 and 6082 alloys, whereas the 6111 alloy exhibited a more homogeneous orientation distribution. In addition, TEM observations showed finer and more uniformly distributed nanoscale features in the 6111 alloy, which supports its reduced sensitivity to the loading direction.

## 4. Discussion

The increasing incorporation of post-consumer aluminum scrap into 6xxx series alloys poses well-known challenges related to chemical variability, impurity accumulation and melt cleanliness, which can directly affect the extrusion stability and mechanical reliability. The present study demonstrates that these challenges can be effectively addressed when the recycling route is rigorously controlled, enabling high scrap ratios without compromising product quality or performance.

A key enabler of this controlled route is the use of advanced sensor-based sorting prior to melting. The MULTI-PICK technology was employed to stabilize the feedstock composition by separating alloy families and limiting impurity fluctuations at the earliest stage of the process. From an industrial standpoint, such pre-sorting is critical for reducing downstream variability and melt treatment intensity. The effectiveness of this approach is confirmed by the hydrogen, PREFIL and ultrasonic inspection results, which collectively indicate a clean melt and defect-free billets that are suitable for extrusion. The combined ultrasonic inspection results and feedstock information therefore establish an essential quality baseline, ensuring that the subsequent microstructural and mechanical evaluations reflect intrinsic alloy behavior, rather than casting-related defects [16,17].

Microstructural characterization further supports the effectiveness of this integrated recycling strategy. Optical microscopy shows that homogenization significantly reduces microsegregation and promotes a more uniform matrix structure, even in alloys produced with high post-consumer scrap content. Importantly, no detrimental needle-like features that are typically associated with poor melt quality were observed after homogenization in the scrap-derived 6063 and 6082 alloys, which is consistent with the diffusion-controlled homogenization mechanisms reported for industrial 6xxx billets [18,26]. These observations indicate that the elevated Fe levels that are inherent to scrap-rich feedstocks can be effectively managed through appropriate alloy design and thermal processing.

SEM, TEM and EBSD analyses reveal clear differences in microstructural uniformity and deformation characteristics among the investigated alloys. While the scrap-based 6063 and 6082 alloys exhibit more pronounced extrusion-induced texture and heterogeneous microstructural features, the CRM-lean 6111 alloy displays a finer and more uniformly distributed nanoscale microstructural state, together with a smoother orientation distribution. Rather than attributing these observations to specific phase identities, the results indicate differences in microstructural homogeneity and grain boundary character, which are known to influence strain accommodation and deformation behavior in extruded aluminum alloys [26].

These microstructural differences are directly reflected in the anisotropic tensile response. The stronger orientation dependence observed in the 6063 and 6082 alloys correlates with their more pronounced texture and heterogeneous boundary distributions, whereas the 6111 alloy exhibits reduced strength anisotropy and a more uniform mechanical response across extrusion directions. This behavior is consistent with previous studies showing that refined and homogeneous microstructural features promote more uniform plastic deformation and mitigate orientation sensitivity in Al–Mg–Si alloys [26,27]. Recent operando investigations reported in Acta Materialia further support this interpretation, demonstrating that a refined and spatially uniform distribution of intermetallic-containing features enhances deformation homogeneity and delays strain localization in recycled aluminum systems with complex chemistry [25].

From a broader industrial and sustainability perspective, the present results align with recent reports on high-recycled aluminum systems, including aerospace-grade alloys, where careful impurity control enables high mechanical performance despite significant scrap input [9]. Unlike studies focused on a single alloy or laboratory-scale processing, the present work demonstrates a multi-alloy, full-scale extrusion validation framework. The combination of MULTI-PICK sorting, controlled melt treatment, homogenization and extrusion provides a scalable pathway for producing automotive-grade 6xxx alloys with a high recycled content and reduced dependence on primary aluminum.

Overall, this study confirms that highly recycled 6xxx aluminum alloys can reliably replace primary feedstock in demanding automotive applications when an integrated process strategy is applied. Moreover, the development of a CRM-lean 6111 alloy highlights how recycling and alloy design can act synergistically to reduce critical raw material usage while maintaining mechanical performance. These findings contribute directly to sustainable, low-carbon aluminum processing strategies and support circular-economy objectives that are in line with the European Green Deal.

## 5. Conclusions

This study demonstrates that high post-consumer scrap content can be successfully integrated into 6xxx aluminum alloys when advanced sorting, melt quality control and optimized thermomechanical processing are applied in a coordinated manner. Similar process-integrated recycling strategies have recently been identified as key enablers for sustainable aluminum production and recycling-tolerant alloy design [28]. Hydrogen and PREFIL analyses confirmed that appropriate refining practices ensure that melt cleanliness is compatible with stable extrusion processing, while ultrasonic inspection and homogenization effectively minimize casting-related defects and microsegregation, which is in agreement with the previous findings on the importance of melt quality control in recycled aluminum systems [16,17].

Microstructural characterization using OM, SEM/EDS, TEM and EBSD revealed that scrap-derived 6063 and 6082 alloys develop a stable and uniform microstructural state after homogenization, without exhibiting the detrimental microstructural features that are typically associated with poor melt quality. Recent studies have emphasized that the microstructural uniformity and grain-boundary character play a decisive role in governing deformation behavior and reliability in recycled aluminum alloys. Differences in the crystallographic orientations and grain boundary distributions observed among the investigated alloys are consistent with reports highlighting the influence of texture and boundary character on extrusion behavior and mechanical anisotropy [29].

Mechanical testing confirmed that both high-recycled 6xxx alloys and the CRM-lean 6111 alloy meet the mechanical requirements for automotive structural applications, with anisotropy levels remaining within the ranges that are commonly reported for extruded Al–Mg–Si alloys [26]. The reduced strength anisotropy observed in the 6111 alloy highlights the potential of combining alloy design strategies with recycling approaches to reduce critical raw material dependency while maintaining reliable mechanical performance, as also discussed in the recent literature on recycling-compatible alloy development [28,29].

Overall, the results confirm that highly recycled 6xxx aluminum alloys can effectively replace primary-based feedstock in automotive applications when advanced sensor-based sorting technologies, controlled melt treatment, homogenization and extrusion routes are properly integrated. In this context, industrial-scale sensor-based sorting solutions, such as MULTI-PICK, enable feedstock consistency and process stability, which are recognized as essential prerequisites for scalable and economically viable aluminum recycling routes [10,28]. These findings support the ongoing efforts toward sustainable, low-carbon aluminum production and contribute to the broader implementation of circular-economy strategies in metallurgical processing.

## Figures and Tables

**Table 1:** Feedstock contents for newly developed 6111 and high-recycling-content 6063 and 6082 alloys.

	6063	6082	6111
MULTI-PICK Scrap	16%	34%	0
6063 Post Consumer Scrap	54%	12%	0
6082 Post Consumer Scrap	0	54%	0
6063 Pre-Consumer Scrap	30%	0%	0
Primary Feedstock	0	0	100%

**Table 2:** Chemical compositions of the newly developed 6111 and high-recycling-content 6063 and 6082 alloys.

	Si	Fe	Cu	Mn	Mg	Cr	Zn	Ti
6111	0.66	0.15	0.9	0.25	0.72	0.001	0.01	0.01
6063	0.55	0.23	0.02	0.06	0.55	0.01	0.05	0.02
6082	0.88	0.25	0.75	0.62	0.86	0.06	0.04	0.016

**Table 3:** Elemental percentages of the area and spot EDS analyses.

	6063 Profile		6111 Profile		6082 Profile	
Element	Area 1	Spot 1	Area 1	Spot 1	Area 1	Spot 1
Al	64.74	95.97	61.79	98.96	98.32	79.73
Fe	25.02	0.5	19.72	0.21	0.16	8.83
Si	8.61	0.82	5.45	0.01	0.03	3.33
Mg	1.26	0.88	0.5	0.32	0.49	0.99
Mn	0.37	0.27	9.52	0.31	0.43	6.62
Cu		0.89	3.02		0.32	0.5
Zn		0.66		0.19	0.25	

**Table 4:** EDS analysis of 6063 profile’s selected zones, with respect to elemental weight and atomic percentages.

	Weight %						Atomic %					
	Mg	Al	Si	Mn	Fe	Cu	Mg	Al	Si	Mn	Fe	Cu
O_1_	2.93	84.16	3.65	4.79	3.42	1.03	3.41	88.24	3.67	2.46	1.73	0.45
O_2_	4.02	86.44	2.82	3.05	2.56	1.08	4.61	89.27	2.82	1.54	1.28	0.47
O_3_	2.1	95.22	1.5		1.16		2.34	95.63	1.45		0.56	
Area 1	0.06	98.75	0.26	0.2	0.23	0.46	0.07	99.24	0.26	0.2	0.23	0.46

**Table 5:** EDS analysis of 6111 profile’s selected zones, with respect to elemental weight and atomic percentages.

	Weight %						Atomic %					
	Mg	Al	Si	Mn	Fe	Cu	Mg	Al	Si	Mn	Fe	Cu
Point 1	1.86	86.04	2.21	8.24	0.65	0.96	2.18	90.54	2.24	4.26	0.33	0.43
Point 2	4.17	84.34	8.4	0.46	0.1	2.5	4.71	85.72	8.2	0.23	0.05	1.07
Area 1	1.28	96.61	0.37			1.72	1.44	97.45	0.36			0.73

**Table 6:** EDS analysis of 6082 profile’s selected zones, with respect to elemental weight and atomic percentages.

	Weight %							Atomic %						
	Mg	Al	Si	Cr	Mn	Fe	Cu	Mg	Al	Si	Cr	Mn	Fe	Cu
Point 1	0.59	84.65	4.09	1.26	8.74	0.64		0.7	89.57	4.16	0.69	4.54	0.32	
Point 2		84.31	3.82	1.74	9.64	0.46			89.82	3.91	0.96	5.04	0.24	
Point 3	3.64	92.64	1.63	0.09	1.77	0.05	0.15	4.07	93.32	1.57	0.05	0.87	0.02	0.06
Point 4	0.86	89.51	2.8	0.72	5.51	0.58		0.98	92.72	2.79	0.39	2.8	0.29	

**Table 7:** Tensile test results of 6111, 6063 and 6082 alloy profiles, with respect to 0°, 45° and 90° of extrusion direction.

Alloy	Angle	Rp0.2 [MPa] (95% CI)	Rm [MPa] (95% CI)	A (%) (95% CI)
6063	0°	244 ± 6	270 ± 3	13.6 ± 1.4
	45°	238 ± 5	257 ± 6	5.95 ± 1.8
	90°	281 ± 4	321 ± 3	8.9 ± 1.3
6111	0°	306 ± 6	326 ± 4	10 ± 1.8
	45°	302 ± 5	325 ± 6	5.3 ± 1.6
	90°	303 ± 5	324 ± 7	7.6 ± 2.0
6082	0°	267 ± 5	293 ± 4	15.3 ± 1.6
	45°	264 ± 4	287 ± 5	9.1 ± 1.9
	90°	276 ± 3	300 ± 4	9.2 ± 2.1

## Data Availability

The original contributions presented in this study are included in the article. Further inquiries can be directed to the corresponding author.
